# Case series: Psychosocial challenges of female youth within the Irish travelling community

**DOI:** 10.1192/j.eurpsy.2022.839

**Published:** 2022-09-01

**Authors:** E. O’ Neill, N. Abdul-Razak, Z. Anastasova, C. O’ Callaghan

**Affiliations:** HSE, Psychiatry, Cork, Ireland

**Keywords:** Irish travelling community, mental illness within the travelling community, women within the Irish t

## Abstract

**Introduction:**

The Irish traveller community are an ethnic minority group known for their distinct identity. Although this group has its roots in Ireland, they are marginalised and discriminated against by every part of Irish society. Adolescent females encounter particular difficulties within the expectations of this community. They encounter specific issues including mental illness, sexual stigma and limitations to the role of women.

**Objectives:**

Explore the vulnerabilities of young women within the irish travelling community.

**Methods:**

Literature review and case series using three cases.

**Results:**

Patient A is a nineteen-year-old girl known with a history of overdose and depression. Significant triggers for her mental illness are linked to familial disharmony and sexual assault. Patient B is seventeen years old and was referred for CAMHS inpatient admission following overdose. She has a background of sexual assault and drug misuse. Patient C is fifteen years old and was admitted to a CAMHS unit following a hanging attempt. Her suicide attempt was triggered by chronic bullying, grief and sexual assault.

**Conclusions:**

Young women in the travelling community are estimated to be twice as likely to suffer mental health issues as compared to men. They are primed to follow a culture where the main events in life are centred around training for marriage and child rearing. In this world of ethical practice and focus on women’s rights, females in such communities can feel conflicted between their identity, heritage and their position in the world. Adolescents within the travelling community should be monitored with consciousness given to their particular risk factors.

**Disclosure:**

No significant relationships.

